# Self-Reported Physical Activity: Its Correlates and Relationship with Health-Related Quality of Life in a Large Cohort of Colorectal Cancer Survivors

**DOI:** 10.1371/journal.pone.0036164

**Published:** 2012-05-02

**Authors:** Laurien M. Buffart, Melissa S. Y. Thong, Goof Schep, Mai J. M. Chinapaw, Johannes Brug, Lonneke V. van de Poll-Franse

**Affiliations:** 1 Department of Epidemiology and Biostatistics and EMGO Institute for Health and Care Research, VU University Medical Center, Amsterdam, The Netherlands; 2 Center of Research on Psychology in Somatic Diseases (CoRPS), Tilburg University, Tilburg, The Netherlands; 3 Comprehensive Cancer Centre South, Eindhoven, The Netherlands; 4 Department of Sports Medicine, Maxima Medical Center Veldhoven, Veldhoven, The Netherlands; 5 Department of Public and Occupational Health and EMGO Institute for Health and Care Research, VU University Medical Center, Amsterdam, The Netherlands; Universidad Europea de Madrid, Spain

## Abstract

**Background:**

Physical activity (PA) is suggested to be an important non-pharmacologic means to improve health-related outcomes among cancer survivors. We aimed to describe the PA level, its correlates, and association with health-related quality of life (HRQoL) in colorectal cancer (CRC) survivors.

**Methods:**

CRC survivors identified from the Eindhoven Cancer Registry treated between 1998 and 2007 were included. Survivors completed validated questionnaires on PA, distress, fatigue, and HRQoL. Moderate-to-vigorous physical activity (MVPA) levels were calculated by summing the time spent on walking, bicycling, gardening and sports (≥3 MET). Multiple linear regression analyses were conducted to study which socio-demographic and clinical factors were associated with MVPA. Furthermore, we examined associations between MVPA and physical and mental HRQoL, and whether these associations were mediated by fatigue and distress.

**Results:**

Cross-sectional data of 1371 survivors (response: 82%) were analysed. Participants were 69.5 (SD 9.7) years old, 56% were male, and survival duration was 3.9 (SD 2.5) years. Participants self-reported on average 95.5 (SD 80.3) min on MVPA per day. Younger age, male sex, being employed, non-smoking, lower BMI, colon cancer (vs. rectal cancer), chemotherapy treatment and having no co-morbidities were associated with higher MVPA (p<0.05). MVPA was positively associated with physical HRQoL (regression coefficient of total association (c) = 0.030; se = 0.004) after adjusting for socio-demographic and clinical factors. Fatigue mediated this association between MVPA and physical HRQoL (44% mediated). The association between MVPA and mental HRQoL was not statistically significant after adjusting for socio-demographic and cancer-related factors (c = 0.005; se = 0.004).

**Conclusion:**

In CRC survivors, clinical factors including the absence of co-morbidity, tumour site and chemotherapy treatment were associated with higher MVPA, in addition to several socio-demographic factors. Higher MVPA was associated with higher physical HRQoL but not with mental HRQoL. Fatigue and distress mediated the association between MVPA and HRQoL.

## Introduction

Colorectal cancer (CRC) is one of the most common cancers worldwide. In the Netherlands, it is the second most common cancer, with 12,000 (54% male) new cases diagnosed in 2009 [1]. Recent advances in early detection and treatment have led to improved survival rates. In the Netherlands, the current 5-year survival rate for CRC is over 50% [1]. Many patients face psychosocial and physical problems during and after cancer treatment, including fatigue, increased risk of distress, and reduced physical fitness and physical function [2], [3]. These long-term sequelae can affect the patient's health-related quality of life (HRQoL) [4]–[6], and there is empirical evidence of an association between HRQoL and survival [7]. There is need for effective methods to manage treatment side effects and to improve HRQoL in cancer patients and survivors [8].

Physical activity (PA) is a modifiable behaviour, and has been proposed as an important non-pharmacologic means to improve health-related outcomes among cancer patients and survivors. Regular PA may influence health outcomes after a cancer diagnosis [9], [10]. Recent data suggests that regular PA may reduce the likelihood of cancer recurrence and mortality among CRC patients [11]–[14]. In addition, a number of previous studies found PA to be beneficial for HRQoL in CRC patients and survivors [15]–[18]. From previous studies in cancer patients, it is also known that PA interventions have beneficial effects on fatigue and distress [19]–[23].

With respect to the development of future interventions to improve PA and HRQoL in CRC survivors, it is important to obtain insight in the level of PA and its associated socio-demographic and cancer-related factors. Furthermore, we need a better understanding of the association between PA and HRQoL; whether this association is direct or via other somatic, social, and/or psychological factors. More insight into how PA is associated with HRQoL is necessary for the systematic progression of research in this field and to better inform future PA interventions for CRC survivors. In a small sample of 27 women who were treated with chemotherapy for breast cancer, Schwartz [24] found that the effect of exercise on quality of life was mediated by fatigue. It is unclear whether this could also be the case in CRC survivors.

The aim of this study was to describe the PA level in a large group of Dutch CRC survivors, and to identify which demographic and cancer-related factors were associated with PA. The second aim was to study whether PA was associated with HRQoL, and whether this association was mediated by fatigue and distress.

## Methods

### Setting and participant recruitment

**Figure 1:**
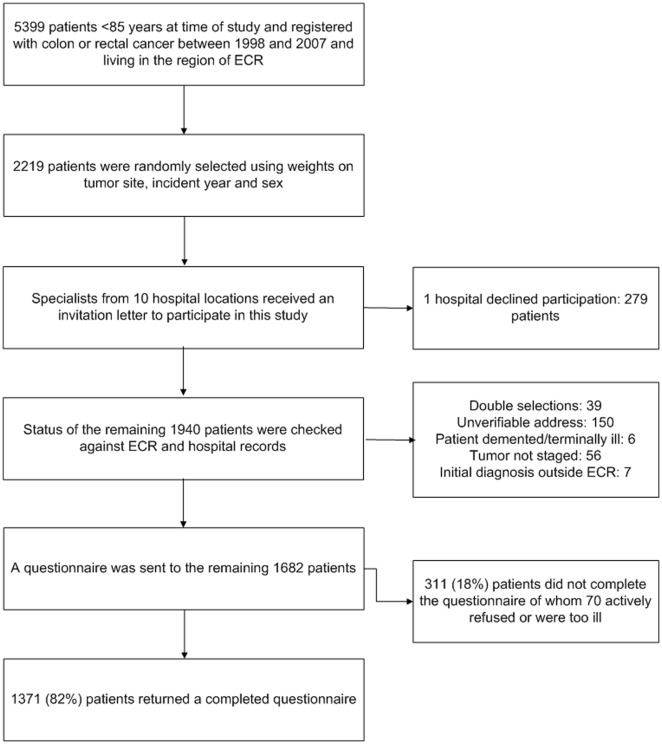
Flow-chart of the data collection process. ^1^ ECR: Eindhoven Cancer Registry ^2^ CBG: Central Bureau of Genealogy.

The Eindhoven Cancer Registry (ECR) records data on all newly diagnosed cancer patients in the southern part of the Netherlands, an area with 2.4 million inhabitants, 10 hospitals, and two large radiotherapy institutes [25]. Individuals diagnosed with colon or rectal cancer in the period 1998–2007, as registered in the ECR, were eligible. Patients who had died, according to the ECR and the Central Bureau for Genealogy, which records all deaths via the Dutch civil municipal registries and hospital records, were excluded. From the potential sample of 5399 survivors, a weighted random selection of 2219 survivors based on year of diagnosis and sex was made (Figure 1). The weights were derived from the distribution of colon and rectal cancer survivors in the general population [26], [27]. Survivors with fewer years since diagnosis were oversampled for inclusion in future follow-up assessments. After excluding survivors for reasons shown in Figure 1, data collection started in January 2009. The Medical Ethics Committee of the Maxima Medical Center approved this study.

Eligible survivors were informed about the study by a letter from their (former)-attending surgeons. The letter explained that by completing and returning the enclosed questionnaire survivors consented to participate in the study and agreed to the linkage of the questionnaire data with their disease history in the ECR. Survivors were reassured that refusal to participate had no consequences for their follow-up care or treatment. Non-respondents were sent a reminder letter with a new copy of the questionnaire within two months.

Raw data from colorectal cancer survivors analysed for this study are available for non-commercial scientific research, subject to study question, privacy and confidentiality restrictions, and registration (www.profilesregistry.nl) [28].

### Measures

PA was assessed with questions derived from the validated European Prospective Investigation into Cancer (EPIC) Physical Activity Questionnaire [29]. Compared with accelerometers, the EPIC physical activity questionnaire has acceptable measurement characteristics for ranking participants according to total PA level [30]. Participants were asked how much time they spend on the following activities (average number of hours per week, in summer and winter separately): walking, bicycling, gardening, housekeeping, and sports. Six separate sports could be specified. The mean number of hours of PA per week in summer and winter were computed. To include an estimate of intensity, metabolic equivalent intensity values (MET value: 1 MET = 4.184 kJ/kg body weight/h) were assigned to each activity, according to the compendium of physical activities [31], [32]: 3.5 MET for walking (code 17250, walking for pleasure), 4 MET for bicycling (code 01010, bicycling general, for pleasure), 5 MET for gardening (general gardening, code 08245), 3.5 MET for housekeeping (code 05030, cleaning house general), and varying intensities for the different sports. Total PA was calculated by summing hours/week of all activities. The duration of moderate to vigorous physical activity (MVPA) was assessed as time (min/wk) spent on walking, bicycling, gardening and sports (≥3 MET), excluding housekeeping and light intensity sports (<3 MET). The level of MVPA was also expressed in kJ/kg body weight/day.

Patients' socio-demographic and clinical information were available from the ECR. The ECR routinely collects data on tumour characteristics, including date of diagnosis, tumour grade and stage according to the 6^th^ edition of the Tumour-Node-Metastasis clinical classification [33], treatment, and patient demographics at the time of diagnosis.

Self-reported co-morbidity at the time of questionnaire completion was categorized according to an adapted Self-administered Co-morbidity Questionnaire (SCQ) [34]. Socioeconomic status was determined by the investigator using an indicator developed by Statistics Netherlands based on individual fiscal data from the year 2000 on the economic value of the home and household income, and provided as aggregate level for each postal code (average 17 households) [35], which were then categorized into tertiles. Self-reported height and weight, marital status, educational level, employment status and smoking status were also assessed.

Emotional distress was assessed with the 14-item Hospital Anxiety and Depression Scale (HADS) [36], [37]. The HADS has been validated for the Dutch population [38], and demonstrated adequate psychometric properties for cancer patients [39]. It yields a total score and separate scale scores for anxiety and depression. Possible anxiety and depression was indicated by a score ≥8 on the respective subscales.

Fatigue was assessed by the Fatigue Assessment Scale (FAS), which has been validated for the Dutch population [40], [41]. This questionnaire consists of 10 statements on fatigue, on a scale from 1 (never) to 5 (always). Total fatigue score was calculated by summing the scores of the individual items. Fatigue was indicated by a total score ≥22.

HRQoL was assessed with the Dutch version of the Short Form-36 (SF-36) questionnaire [42]. The SF-36 consists of eight subscales: physical function, role limitations due to physical health, bodily pain, general health perceptions, vitality, social function, role limitations due to emotional health. The subscales were further combined into the physical (PCS) and mental (MCS) component summary scores. All scales were linearly converted to a 0–100 scale according to standard scoring procedures, with higher scores indicating better HRQoL.

### Statistical analysis

Descriptive statistics were presented as mean (standard deviation, SD) or as numbers (percentages). Differences in demographic and clinical data between participants and non-participants were determined using an independent T-test or Chi-square test. Data on PA were presented as mean (SD) for the separate activities, as well as for total PA and MVPA. Impossible high values (≥56 h/week) of walking (n = 3), cycling (n = 2), gardening (n = 1), housekeeping (n = 9), sports (n = 1), or MVPA (n = 8) were imputed by using the 95^th^ percentiles.

First, univariate linear regression analyses were conducted to study the association between independent socio-demographic and clinical factors and MVPA level. Subsequently, a multiple linear regression model was constructed including all variables. Due to high collinearity (r>0.75) between tumour site and radiotherapy treatment, we did not include radiotherapy in the multivariate linear regression analysis.

We also performed univariate and multiple linear regression analyses to study the association between MVPA and HRQoL (PCS and MCS score). To study whether fatigue (total FAS score) and distress (total HADS score) were mediators in the association between PA and HRQoL, we conducted a series of linear regression analyses according to the product of coefficient method described by MacKinnon [43]. First, we calculated the association between MVPA and HRQoL (total association, c-path). Second, we calculated the association between the MVPA and the potential mediator (fatigue or distress) (a-path). Third, we calculated the association between the potential mediator and HRQoL, controlled for MVPA level (b-path). The final regression model also provided estimates for the direct association between MVPA and HRQoL (c′-path). All regression models were adjusted for relevant socio-demographic and cancer-related characteristics. The product of coefficients (a×b) provided an estimate of the relative strength of the mediation effect [43]. The proportion mediated was estimated by dividing the mediation effect by the total association ((a×b)/c). A bootstrapping method was used to calculate the bias corrected confidence intervals (CI) around the mediated and direct associations using the SPSS macro suggested by Preacher and Hayes [44]. The criterion of the mediation framework MacKinnon [43] suggests, in contrast to Baron and Kenny [45], that the potential mediation effect should also be analysed even if the c-path (total association between the independent and dependent variable) is not significant. If the total association is not significant, mediation can occur when a suppression effect is present [46], i.e. when the direct and mediating effect of the independent variable on the outcome variable have opposite signs. In this case, the proportion mediated is not informative. Finally, a multiple mediation analysis was conducted including both fatigue and distress as mediators in the models.

## Results

In total, 1371 (82%) CRC survivors returned the questionnaires, of whom 56% were men. Mean (SD) age of the CRC survivors was 69.5 (9.7) years, of which 69% were aged 65 years or older. Of these survivors, 66% were diagnosed with colon cancer and 34% with rectal cancer.

Study participants were on average 2.1 years younger, more often male and diagnosed with rectal cancer compared to non-participants (p<0.05), see Table 1. Other socio-demographic and clinical characteristics including socioeconomic status, survival duration, tumour site and stage, and type of treatment did not differ between participants and non-participants.

**Table 1:** Descriptive statistics of the study sample.

Socio-demographic factors	Participants (n = 1371)	Non-participants (n = 311)
Age, mean (SD) years*	69.5 (9.7)	71.6 (10.0)
Gender, n (%) males*	774 (56)	141 (45)
Married/partner, n (%)	984 (74)	
Socioeconomic status, n (%)		
Low	510 (22)	101 (32)
Medium	530 (40)	129 (41)
High	294 (38)	73 (23)
Employment, n (%)		
Employed	195 (15)	
Retired/unemployed	1107 (85)	
Education, n (%) high	254 (19)	
Current smoker, n (%) yes	145 (11)	
BMI, mean (SD) kg/m^2^	26.7 (4.3)	
Overweight, n (%)	619 (48)	
Obese, n (%)	205 (16)	
**Cancer-related factors**		
Survival, mean (SD) years	3.9 (2.5)	3.9 (2.5)
≥5 years, n (%)	353 (26)	85 (27)
Tumour site, n (%)*		
Colon cancer	908 (66)	227 (73)
Rectal cancer	463 (34)	84 (27)
Tumour stage, n (%)		
1	387 (28)	83 (27)
2	528 (39)	134 (43)
3	385 (28)	86 (28)
4	71 (5)	8 (2)
Type of treatment, n (%)		
Surgery	731 (53)	195 (63)
Radiotherapy	1 (0.1)	0 (0)
Chemotherapy	11 (1)	3 (1)
Surgery + Radiotherapy	271 (20)	43 (14)
Surgery + Chemotherapy	284 (21)	53 (17)
Surgery + Radiotherapy + Chemotherapy	73 (5)	15 (5)
Co-morbidity, n (%) yes	429 (31)	
**Physical Activity (PA)**		
Walking, mean (SD) h/week	5.2 (5.3)	
Cycling, mean (SD) h/week	4.2 (5.5)	
Gardening, mean (SD) h/week	2.4 (3.7)	
Housekeeping, mean (SD) h/week	9.4 (9.8)	
Sports, mean (SD) h/week	4.6 (4.9)	
Total PA, mean (SD) h/week	19.1 (14.7)	
MVPA, mean (SD) min/day	95.5 (80.3)	
MVPA, mean (SD) kJ/kg/d	28.3 (25.7)	
**Psychological factors**		
Distress, HADS score, mean (SD)	9.8 (6.5)	
Fatigue, FAS score, mean (SD)	21.1 (7.3)	
**Health-related quality of life**		
Physical component summary, SF-36, mean (SD)	47.8 (10.9)	
Mental component summary, SF-36, mean (SD)	50.5 (9.5)	

BMI = body mass index; FAS = fatigue assessment scale; HADS = hospital anxiety and depression scale; PA = physical activity; MVPA = recreational moderate to vigorous physical activity; SD = standard deviation; SF 36 = short form 36.

Total PA is the sum of walking, cycling, gardening, housekeeping and sports; MVPA excludes housekeeping and light intensity sports (<3 METs).

* p<0.05 for differences between participants and non-participants.

Of all participants, 34% engaged in sports. Participants spent on average 19.1 (14.7) h/week on total PA (Table 1). When excluding household activities and light intensity sports (<3 MET), participants spent on average 95.5 (80.3) min/day on MVPA, corresponding to 28.3 (25.7) kJ/kg/d. Anxiety (HADS-A≥8), depression (HADS-D≥8) and fatigue (FAS≥22) was present in 21%, 22%, and 42% of participants respectively.

### Correlates of physical activity

Univariate regression analysis showed that CRC survivors who were younger, male, had a partner, were treated with chemotherapy and had no co-morbidities had higher levels of MVPA (p<0.05; Table 2). Multivariate analysis showed that CRC survivors who were younger, male, employed, non-smoking, had lower BMI, colon cancer (instead of rectal cancer), chemotherapy treatment and no co-morbidities had higher levels of MVPA (p<0.05). When radiotherapy was added to the multivariate model instead of tumour site, results showed that radiotherapy treatment was significantly associated with lower levels of MVPA (β = −11.82, 95% CI = −22.38; −1.27).

**Table 2:** Socio-demographic and clinical correlates of moderate-vigorous physical activity (min/day).

	Univariate			Multivariate		
Socio-demographic factors	B	95%CI	p	B	95%CI	p
Age (years)	−0.66	−1.12; −0.20	0.005	**−1.05**	**−1.69; −0.42**	**0.001**
Gender	−26.82	−35.68;−17.97	<0.001	**−26.89**	**−36.97; −16.81**	**<0.001**
Partner	23.96	13.55; 34.37	<0.001	9.80	−1.68; 21.27	0.09
High education	7.88	−3.30; 19.05	0.17	−1.34	−13.09; 10.41	0.82
Employed	−0.21	−12.59; 12.16	0.97	**23.69**	**7.82; 39.56**	**0.003**
Current smoker	−12.24	−23.42; 1.95	0.09	**−18.21**	**−33.06; −3.36**	**0.02**
BMI (kg/m^2^)	−0.67	−1.75; 0.42	0.23	**−1.43**	**−2.58; −0.27**	**0.02**
**Clinical factors**						
Survival (years)	−0.60	−2.41; 1.21	0.52	0.07	−1.83; 1.98	0.94
Tumour site	−1.33	−10.67; 8.02	0.78	**−10.12**	**−20.01; −0.22**	**0.05**
Tumour stage	1.45	−7.97; 10.86	0.76	−8.68	−21.46; 4.11	0.18
Chemotherapy	12.86	2.81; 22.89	0.01	**15.29**	**1.37; 29.20**	**0.03**
Radiotherapy	−3.87	−13.89; 6.14	0.45	-	-	-
Co-morbidity	−19.84	−29.50; −10.18	<0.001	**−12.31**	**−22.85; −1.77**	**0.02**

Gender (0 = male; 1 = female), partner (1 = yes; 0 = no), education (1 = yes; 0 = no), employed (1 = yes; 0 = no), current smoker (1 = yes; 0 = no); tumour site (0 = colon; 1 = rectal), tumour stage (1–2 = 0; 3–4 = 1); chemotherapy (1 = yes; 0 = no), radiotherapy (1 = yes; 0 = no), co-morbidity (1 = moderate/severe; 0 = none/mild).

Significant associations in the multivariate analysis are presented in bold.

### Association between physical activity and health-related quality of life

MVPA was positively associated with PCS (Table 3), also after adjusting for socio-demographic and clinical factors (c = 0.030; se = 0.004). CRC survivors with higher levels of MVPA reported significantly lower fatigue (a = −0.017; se = 0.003) and distress (a = −0.007; se = 0.003), and lower fatigue (b = −0.782; se = 0.036) and lower distress (b = −0.559; se = 0.046) were associated with higher PCS (Table 3). Consequently, fatigue and distress both mediated the association between MVPA and PCS; fatigue mediated 44% of the association between total PA and PCS, and distress mediated 13%. However, in the multiple mediation analyses, the association between distress and PCS became insignificant (b = 0.011; se = 0.053). This indicates that fatigue is a stronger mediator than distress. After including both fatigue and distress as mediators in the regression model, the direct association between MVPA and PCS remained significant (c′ = 0.017; se = 0.003), indicating partial mediation.

**Table 3:** The association between MVPA and HRQoL and potential mediators fatigue and distress.

Outcome variable	Univariate association between MVPA and HRQoL	Total association between MVPA and HRQoL	Mediator Variable	Direct association between MVPA and HRQoL adjusted for mediator	Association between MVPA and Mediator	Association between mediator and HRQoL, adjusted for MVPA	Mediation effect
		(path c)		(path c′)	(path a)	(path b)	(a×b)
	*Estimate (SE)*	*Estimate (SE)*		*Estimate (SE)*	*Estimate (SE)*	*Estimate (SE)*	*Estimate (95% CI)*
PCS	0.036 (0.004)*	0.030 (0.004)*					
			Fatigue	0.017 (0.003)*	−0.017 (0.003)*	−0.782 (0.036)*	0.013 (0.008; 0.018)*
			Distress	0.026 (0.004)*	−0.007 (0.003)*	−0.559 (0.046)*	0.004 (0.001; 0.007)*
MCS	0.006 (0.004)#	0.005 (0.004)					
			Fatigue	−0.008 (0.003)*	−0.017 (0.003)*	−0.798 (0.034)*	0.014 (0.009; 0.019)*
			Distress	−0.002 (0.003)	−0.007 (0.003)#	−1.001 (0.035)*	0.007 (0.002; 0.012)*

* p<0.05;

# 0.10<p<0.05.

All multivariate models are adjusted for age, gender, partner, employment, education, smoking, body mass index, survival, tumour site and stage, chemotherapy and co-morbidity;

CI = confidence interval; HRQoL = health-related quality of life; MCS = mental component summary; MVPA = moderate-vigorous physical activity; PCS = physical component summary; SE = Standard error.

MVPA was not significantly associated with MCS after adjusting for socio-demographic and clinical factors (c = 0.005; se = 0.004, Table 3). Lower fatigue (b = −0.798; se = 0.034) and lower distress (b = −1.001; se = 0.035) were significantly associated with higher MCS. Consequently, fatigue (a×b = 0.014; se = 0.019) and distress (a×b = 0.007; se = 0.012) were significant mediators of the association between MVPA and MCS (Table 3). In the multiple mediation analysis, both mediators remained significant. After including fatigue and distress as mediators in the regression model, the direct association between MVPA and MCS became negative (c′ = −0.006; se = 0.003), indicating a suppression effect.

## Discussion

This cross-sectional study reports on the self-reported PA levels of a large group of Dutch CRC survivors, on socio-demographic and clinical factors associated with MVPA, and on the association between PA and HRQoL. This study showed that Dutch CRC survivors reported spending 19.1 (SD 14.7) h/week on total PA and 11.1 (SD 9.3) h/week on MVPA. Using a similar PA questionnaire, Voskuil et al. [47] showed that 2 years after diagnosis, Dutch breast cancer survivors reported spending 24.2 (14.7) h/week on total PA, and 9.8 (7.4) h/week on MVPA. Differences between breast and colon cancer might be related to gender. Females generally spend more time on housekeeping, compared to males, showing higher levels of total PA. Our results showed that male CRC survivors spent 27 min more on MVPA compared to females. An Australian survey among CRC survivors observed that CRC survivors were more likely to be insufficiently active and inactive compared to a non-cancer population [48]. In contrast, the levels of MPVA of CRC survivors in our study seem comparable to a reference sample of Dutch people aged 65 years and older, who reported spending on average 90 min/day on MVPA [49]. However, they used a different questionnaire which hampers direct comparison of MVPA levels. Future studies using a similar, and preferably objective measure to assess MVPA are needed to directly compare MVPA levels of CRS survivors with the general population. Longitudinal studies are also needed to explore trends in PA levels over time after a cancer diagnosis and treatment.

### Correlates of physical activity

Our results indicate that higher levels of MVPA are more likely among CRC survivors who were younger, male, employed, non-smoking, had lower BMI, were diagnosed with colon rather than rectal cancer, treated with chemotherapy, and had no co-morbidities. Comparably, Speed-Andrews et al. [50] found a similar pattern of correlates, with higher education and a non-married status as additional significant correlates in a population of Canadian CRC survivors.

Associations between PA and socio-demographic factors such as age, gender, employment status, smoking and BMI have also been reported for general –non-cancer- populations [51], [52]. It is important to acknowledge that for cancer patients, additional clinical variables appear to be important for MVPA. Results of the present study showed that CRC survivors who had co-morbidities were less physically active. Comparably, recent results from an Australian cross-sectional study showed that CRC survivors who were overweight and spent more time watching TV were more likely to report co-morbid conditions [53]. Also Speed-Andrews et al. [50] found higher PA in CRC survivors with less co-morbidities. Because of the cross-sectional nature of this study, it is impossible to make causal inferences. Longitudinal studies are needed to study whether physical inactivity results in more co-morbidity in CRC survivors, or whether co-morbidity reduces the likelihood of being physically active.

Furthermore, the results from the present study suggest that rectal cancer survivors are less physically active than colon cancer survivors, and interventions promoting MVPA may need to pay extra attention to rectal cancer patients. As in primary prevention MVPA is associated with reduced risk for colon cancer but not with rectal [54], a possible explanation for the MVPA differences between these tumour types might be a stronger recommendation from the health practitioners in colon cancer which is known to be related to exercise adherence [55]. Unfortunately, we were unable to take this into account in the current study. Differences between rectal and colon cancer patients might also be related to radiotherapy treatment used in rectal cancer patients. Speed-Andrews et al. [50] found lower PA in CRC survivors who were treated with radiotherapy. We found similar results when radiotherapy was added to the model instead of tumour site. However, due to the high correlation between tumour site and radiation therapy, it is unclear whether lower levels of MVPA in rectal cancer patients resulted from radiation therapy or the disease itself.

In contrast to our expectations, we found higher levels of MVPA in patients who were treated with chemotherapy. This association could be explained by selection bias, i.e. patients who were healthier, younger and had less co-morbidities were treated with chemotherapy. Although we adjusted for age and co-morbidities in the analyses, there may still be some residual confounding. It may also be that patients who were treated with chemotherapy received more advice from health practitioners to exercise to counteract effects of chemotherapy. Husson et al. [56] showed that colorectal cancer patients who were treated with chemotherapy received more information about medical tests, treatment and other services, such as rehabilitation, than patients who were not treated with chemotherapy. Whether chemotherapy is indeed related to more intensive rehabilitation and supportive care should be explored in future studies.

### Relationship between physical activity and quality of life

Results of the present study showed that MVPA was associated with physical HRQoL, also after adjusting for socio-demographic and clinical factors. This is in line with other previous observational studies that used a disease specific HRQoL questionnaire [57], [58]. A previous randomized controlled trial evaluating the effects of exercise aiming to improve physical fitness of colorectal cancer patients also showed improvements in HRQoL, but only in patients who increased their cardiovascular fitness over the course of the intervention [17]. MVPA may therefore be recommended to help to improve HRQoL. Future studies are needed to evaluate the optimal intensity and frequency of MVPA. Analysis of the present study further showed that a higher MVPA level was associated with lower levels of fatigue and distress. In addition, our results indicated that fatigue and distress partly mediated the association between MVPA and physical HRQoL, indicating that there was a direct association as well.

No significant total association was found between MVPA and mental HRQoL. However, fatigue mediated the association between MVPA and mental HRQoL, such that higher levels of MVPA were associated with lower fatigue, and subsequently with higher mental HRQoL. After adjusting for mediating factors fatigue and distress, we found the direct association between MPVA and mental HRQoL to be negative. This indicates a suppression effect is present. Other factors influencing mental HRQoL, which we have not measured, may suppress the association. For example, social networks [59] may be important for the mental HRQoL of CRC survivors, however, we did not include this in our study.

### Strengths and Limitations

This is the first study reporting on PA levels of a large representative group of CRC survivors in the Netherlands. Although response rates were high (82%) suggesting good representativeness, participants were younger, included more men, and more rectal cancer patients than the total population of CRC survivors in the Netherlands. Therefore, we may have overestimated the level of MVPA due to age and gender. However, the over-representation of rectal cancer patients may have led to an underestimation of MVPA. Further, two thirds of the patients included in this study were in an early stage of cancer (stage I and II), and therefore, we may have underestimated the psychological symptoms and late treatment-related adverse effects. Nevertheless, the included population reported high levels of fatigue and distress.

The present study is limited by its cross-sectional design, and consequently, no conclusions about causality can be drawn. Fatigue and distress may also be barriers to PA in CRC survivors [60]. Future studies are needed to investigate whether improving MVPA will improve HRQoL, and whether this improvement can be explained by MVPA-induced reductions in fatigue and distress. Another limitation is the use of self-report to assess PA, which is susceptible to recall and social desirability bias [61]. This may have led to an overestimation of PA, and therefore the absolute PA levels should be interpreted with caution.

Finally, we did not include all potential correlates of PA, such as motivational variables and support from the physician, spouses and friends [62], [63].

In conclusion, in CRC survivors, socio-demographic factors and clinical factors were significantly associated with PA. Patients who were younger, male, employed, non-smoking, had lower BMI, colon cancer, chemotherapy treatment and no co-morbidities had higher levels of MVPA. Higher levels of MVPA were directly associated with a higher physical HRQoL. Fatigue and distress mediated the association between MVPA and physical HRQoL, although fatigue was a stronger mediator. The total association between MVPA and mental HRQoL was not significant. However, we found higher MVPA to be associated with lower fatigue and distress, and lower fatigue and distress to be associated with higher mental HRQoL.

## References

[1] IKCnet Dutch Cancer Registration (2012). Survival.. http://www.ikcnet.nl.

[2] Courneya KS, Friedenreich CM (1999). Physical exercise and quality of life following cancer diagnosis: a literature review.. Ann Behav Med.

[3] Courneya KS (2003). Exercise in cancer survivors: an overview of research.. Med Sci Sports Exerc.

[4] Curt GA, Breitbart W, Cella D, Groopman JE, Horning SJ (2000). Impact of cancer-related fatigue on the lives of patients: new findings from the Fatigue Coalition.. Oncologist.

[5] Dimeo FC (2001). Effects of exercise on cancer-related fatigue.. Cancer.

[6] Denlinger CS, Barsevick AM (2009). The challenges of colorectal cancer survivorship.. J Natl Compr Canc Netw.

[7] Montazeri A (2009). Quality of life data as prognostic indicators of survival in cancer patients: an overview of the literature from 1982 to 2008.. Health Qual Life Outcomes.

[8] Speed-Andrews AE, Courneya KS (2009). Effects of exercise on quality of life and prognosis in cancer survivors.. Curr Sports Med Rep.

[9] Irwin ML, Mayne ST (2008). Impact of nutrition and exercise on cancer survival.. Cancer J.

[10] Demark-Wahnefried W, Rock CL, Patrick K, Byers T (2008). Lifestyle interventions to reduce cancer risk and improve outcomes.. Am Fam Physician.

[11] Haydon AM, MacInnis RJ, English DR, Giles GG (2006). Effect of physical activity and body size on survival after diagnosis with colorectal cancer.. Gut.

[12] Meyerhardt JA, Heseltine D, Niedzwiecki D, Hollis D, Saltz LB (2006). Impact of physical activity on cancer recurrence and survival in patients with stage III colon cancer: findings from CALGB 89803.. J Clin Oncol.

[13] Meyerhardt JA, Giovannucci EL, Holmes MD, Chan AT, Chan JA (2006). Physical activity and survival after colorectal cancer diagnosis.. J Clin Oncol.

[14] Meyerhardt JA, Giovannucci EL, Ogino S, Kirkner GJ, Chan AT (2009). Physical activity and male colorectal cancer survival.. Arch Intern Med.

[15] Courneya KS, Friedenreich CM (1997). Relationship between exercise pattern across the cancer experience and current quality of life in colorectal cancer survivors.. J Altern Complement Med.

[16] Courneya KS, Friedenreich CM (1999). Physical exercise and quality of life in postsurgical colorectal cancer patients.. Psychol Health Med.

[17] Courneya KS, Friedenreich CM, Quinney HA, Fields AL, Jones LW (2003). A randomized trial of exercise and quality of life in colorectal cancer survivors.. Eur J Cancer Care (Engl).

[18] Blanchard CM, Baker F, Denniston MM, Courneya KS, Hann DM (2003). Is absolute amount or change in exercise more associated with quality of life in adult cancer survivors?. Prev Med.

[19] Craft LL, Vaniterson EH, Helenowski IB, Rademaker AW, Courneya KS (2012). Exercise Effects on Depressive Symptoms in Cancer Survivors: A Systematic Review and Meta-analysis.. Cancer Epidemiol Biomarkers Prev.

[20] Cramp F, Daniel J (2008). Exercise for the management of cancer-related fatigue in adults.. Cochrane Database Syst Rev.

[21] Duijts SF, Faber MM, Oldenburg HS, van Beurden M, Aaronson NK (2011). Effectiveness of behavioral techniques and physical exercise on psychosocial functioning and health-related quality of life in breast cancer patients and survivors–a meta-analysis.. Psychooncology.

[22] Speck RM, Courneya KS, Masse LC, Duval S, Schmitz KH (2010). An update of controlled physical activity trials in cancer survivors: a systematic review and meta-analysis.. J Cancer Surviv.

[23] Velthuis MJ, Gasi-Idenburg SC, Aufdemkampe G, Wittink HM (2010). The effect of physical exercise on cancer-related fatigue during cancer treatment: a meta-analysis of randomised controlled trials.. Clin Oncol (R Coll Radiol).

[24] Schwartz AL (1999). Fatigue mediates the effects of exercise on quality of life.. Qual Life Res.

[25] Janssen-Heijnen MLG, Louwman WJ, van de Poll-Franse LV, Coebergh JWW (2005). Results of 50 years cancer registry in the South of the Netherlands: 1955–2004 [in Dutch].

[26] Thong MS, Mols F, Lemmens VE, Creemers GJ, Slooter GD (2011). Impact of chemotherapy on health status and symptom burden of colon cancer survivors: a population-based study.. Eur J Cancer.

[27] Thong MS, Mols F, Lemmens VE, Rutten HJ, Roukema JA (2011). Impact of preoperative radiotherapy on general and disease-specific health status of rectal cancer survivors: a population-based study.. Int J Radiat Oncol Biol Phys.

[28] van de Poll-Franse LV, Horevoorts N, van Eenbergen M, Denollet J, Roukema JA (2011). The Patient Reported Outcomes Following Initial treatment and Long term Evaluation of Survivorship registry: scope, rationale and design of an infrastructure for the study of physical and psychosocial outcomes in cancer survivorship cohorts.. Eur J Cancer.

[29] Pols MA, Peeters PH, Ocke MC, Slimani N, Bueno-de-Mesquita HB (1997). Estimation of reproducibility and relative validity of the questions included in the EPIC Physical Activity Questionnaire.. Int J Epidemiol.

[30] Cust AE, Smith BJ, Chau J, van der Ploeg HP, Friedenreich CM (2008). Validity and repeatability of the EPIC physical activity questionnaire: a validation study using accelerometers as an objective measure.. Int J Behav Nutr Phys Act.

[31] Ainsworth BE, Haskell WL, Leon AS, Jacobs DR, Montoye HJ (1993). Compendium of physical activities: classification of energy costs of human physical activities.. Med Sci Sports Exerc.

[32] Ainsworth BE, Haskell WL, Whitt MC, Irwin ML, Swartz AM (2000). Compendium of physical activities: an update of activity codes and MET intensities.. Med Sci Sports Exerc.

[33] Union Internationale Contre le Cancer (UICC) (2002). Classification of Malignant Tumors.

[34] Sangha O, Stucki G, Liang MH, Fossel AH, Katz JN (2003). The Self-Administered Comorbidity Questionnaire: a new method to assess comorbidity for clinical and health services research.. Arthritis Rheum.

[35] van Duijn C, Keij I (2002). Sociaal-economische status indicator op postcode niveau [in Dutch].. Maandstatistiek van de bevolking.

[36] Snaith P, Zigmond AS (1988). Anxiety and depression in general medical settings.. BMJ.

[37] Zigmond AS, Snaith RP (1983). The hospital anxiety and depression scale.. Acta Psychiatr Scand.

[38] Spinhoven P, Ormel J, Sloekers PP, Kempen GI, Speckens AE (1997). A validation study of the Hospital Anxiety and Depression Scale (HADS) in different groups of Dutch subjects.. Psychol Med.

[39] Vodermaier A, Linden W, Siu C (2009). Screening for emotional distress in cancer patients: a systematic review of assessment instruments.. J Natl Cancer Inst.

[40] Michielsen HJ, de Vries J, Van Heck GL (2003). Psychometric qualities of a brief self-rated fatigue measure: The Fatigue Assessment Scale.. J Psychosom Res.

[41] Michielsen HJ, De Vries J, Van Heck GL, van de Vijver FJR, Sijtsma K (2004). Examination of the dimensionality of fatigue. The construction of the Fatigue Assessment Scale (FAS).. Eur J Psychol Assess.

[42] Aaronson NK, Muller M, Cohen PD, Essink-Bot ML, Fekkes M (1998). Translation, validation, and norming of the Dutch language version of the SF-36 Health Survey in community and chronic disease populations.. J Clin Epidemiol.

[43] MacKinnon DP (2008). Introduction to Statistical Mediation Analysis.

[44] Preacher KJ, Hayes AF (2004). SPSS and SAS procedures for estimating indirect effects in simple mediation models.. Behav Res Methods Instrum Comput.

[45] Baron RM, Kenny DA (1986). The moderator-mediator variable distinction in social psychological research: conceptual, strategic, and statistical considerations.. J Pers Soc Psychol.

[46] MacKinnon DP, Krull JL, Lockwood CM (2000). Equivalence of the mediation, confounding and suppression effect.. Prev Sci.

[47] Voskuil DW, van Nes JG, Junggeburt JM, van de Velde CJ, van Leeuwen FE (2010). Maintenance of physical activity and body weight in relation to subsequent quality of life in postmenopausal breast cancer patients.. Ann Oncol.

[48] Hawkes AL, Lynch BM, Youlden DR, Owen N, Aitken JF (2008). Health behaviors of Australian colorectal cancer survivors, compared with noncancer population controls.. Support Care Cancer.

[49] Centraal Bureau voor de Statistiek (2011). Gezondheid, leefstijl, zorggebruik t/m 2009.. http://statline.cbs.nl.

[50] Speed-Andrews AE, Rhodes RE, Blanchard CM, Culos-Reed SN, Friedenreich CM (2011). Medical, demographic and social cognitive correlates of physical activity in a population-based sample of colorectal cancer survivors.. Eur J Cancer Care (Engl).

[51] Salmon J, Owen N, Crawford D, Bauman A, Sallis JF (2003). Physical activity and sedentary behavior: a population-based study of barriers, enjoyment, and preference.. Health Psychol.

[52] Trost SG, Owen N, Bauman AE, Sallis JF, Brown W (2002). Correlates of adults' participation in physical activity: review and update.. Med Sci Sports Exerc.

[53] Hawkes AL, Lynch BM, Owen N, Aitken JF (2011). Lifestyle factors associated concurrently and prospectively with co-morbid cardiovascular disease in a population-based cohort of colorectal cancer survivors.. Eur J Cancer.

[54] Wolin KY, Tuchman H (2011). Physical activity and gastrointestinal cancer prevention.. Recent Results Cancer Res.

[55] Jones LW, Courneya KS, Fairey AS, Mackey JR (2004). Effects of an oncologist's recommendation to exercise on self-reported exercise behavior in newly diagnosed breast cancer survivors: a single-blind, randomized controlled trial.. Ann Behav Med.

[56] Husson O, Thong MS, Mols F, Oerlemans S, Kaptein AA (2012). Illness perceptions in cancer survivors: what is the role of information provision?. Psycho-oncology.

[57] Lynch BM, Cerin E, Owen N, Hawkes AL, Aitken JF (2008). Prospective relationships of physical activity with quality of life among colorectal cancer survivors.. J Clin Oncol.

[58] Grimmett C, Bridgewater J, Steptoe A, Wardle J (2011). Lifestyle and quality of life in colorectal cancer survivors.. Qual Life Res.

[59] Sapp AL, Trentham-Dietz A, Newcomb PA, Hampton JM, Moinpour CM (2003). Social networks and quality of life among female long-term colorectal cancer survivors.. Cancer.

[60] Courneya KS, Friedenreich CM, Quinney HA, Fields AL, Jones LW (2005). A longitudinal study of exercise barriers in colorectal cancer survivors participating in a randomized controlled trial.. Ann Behav Med.

[61] Sallis JF, Saelens BE (2000). Assessment of physical activity by self-report: status, limitations, and future directions.. Res Q Exerc Sport.

[62] Courneya KS, Blanchard CM, Laing DM (2001). Exercise adherence in breast cancer survivors training for a dragon boat race competition: a preliminary investigation.. Psychooncology.

[63] Courneya KS, Friedenreich CM, Reid RD, Gelmon K, Mackey JR (2009). Predictors of follow-up exercise behavior 6 months after a randomized trial of exercise training during breast cancer chemotherapy.. Breast Cancer Res Treat.

